# Qizhu Rougan Granules suppress liver fibrosis by inhibiting the expression of the P2Y14 receptor on hepatic stellate cells

**DOI:** 10.3389/fphar.2024.1528100

**Published:** 2025-01-07

**Authors:** Yujing Tao, Qun Niu, Yuanqian Yao, Kaixin Wang, Haijian Dong, Xin Zhao, Zijian Zeng, Hui Li

**Affiliations:** ^1^ Hospital of Chengdu University of Traditional Chinese Medicine, TCM Hospital of Sichuan Province, Chengdu, Sichuan, China; ^2^ School of Clinical Medicine, Chengdu University of Traditional Chinese Medicine, Chengdu, Sichuan, China

**Keywords:** liver fibrosis, hepatic stellate cells (HSCs), P2Y14, Qizhu-Ruogan-Granules (QZRG), apoptosis

## Abstract

**Introduction:**

Liver fibrosis is a globally prevalent chronic liver disease, often representing the advanced stage of various chronic liver conditions. Despite its widespread occurrence, there is currently no widely accepted or effective treatment for liver fibrosis. However, increasing evidence supports the efficacy of Traditional Chinese Medicine (TCM) in inhibiting the progression of fibrosis. In this study, we explored the effects and potential mechanisms of Qizhu-Ruogan-Granules (QZRG), a formulation from the Affiliated Hospital of the Chengdu University of TCM, on carbon tetrachloride (CCl4)-induced liver fibrosis in mice.

**Methods:**

A total of 40 male C57BL/6J mice were randomly divided into five groups (n = 8 per group), with liver fibrosis induced by injecting 10% CCl_4_ for 15 weeks. From the 7th week onward, QZRG granules were administered orally to the treatment groups at low, medium, and high doses. To assess liver function, serum levels of alanine aminotransferase (ALT), aspartate aminotransferase (AST), and alkaline phosphatase (ALP) were measured. Liver morphology and fibrosis were evaluated using hematoxylin-eosin (H&E) and Masson’s trichrome staining, while gene and protein expression levels were analyzed through quantitative reverse transcription polymerase chain reaction (RT-PCR) and western blot techniques.

**Results:**

The results showed that QZRG granules significantly reduced serum levels of AST, ALT, and ALP in CCl_4_-treated mice, alleviated liver damage, and reduced collagen accumulation. Furthermore, QZRG granules inhibited the expression of apoptosis-related proteins BAX, Caspase9, Caspase8, and Caspase3, while reducing P2Y14 expression in fibrotic liver tissues. Additionally, QZRG granules suppressed the proliferation of activated hepatic stellate cells.

**Conclusion:**

Our findings suggest that QZRG granules may exert anti-fibrotic effects by downregulating P2Y14 expression and effectively slowing the progression of liver fibrosis.

## Graphical Abstract

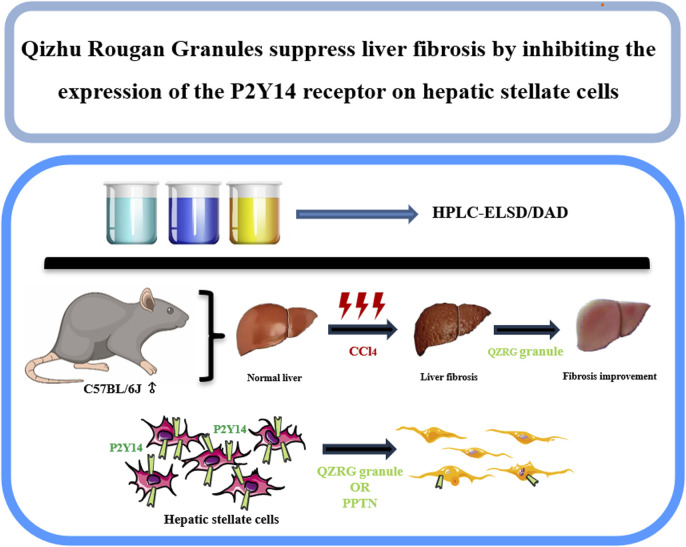

## Highlights

• Qizhu-Ruogan-Granules ameliorates CCl_4_-induced liver fibrosis.
• Continuous activation of hepatic stellate cells is the driving force behind the progression of liver fibrosis.
• Activation of hepatic stellate cells is in a P2Y14-dependent manner.
• Qizhu-Ruogan-Granules downregulated P2Y14 expression by reducing overall apoptosis levels in liver tissues of CCl_4_ model mice.
• Qizhu-Ruogan-Granules promoted apoptosis in activated hepatic stellate cells by inhibiting P2Y14 expression within these cells.

## 1 Introduction

Liver fibrosis is the underlying cause of all end-stage liver disease complications, and its morbidity and mortality rates remain high in many countries due to its widespread prevalence and the lack of effective treatments (Wells, 2009; Udompap et al., 2016; Ciardullo et al., 2021; Kotsiliti, 2023; Man et al., 2023; Åberg et al., 2024; Luo et al., 2024). Increasing evidence suggests that liver fibrosis can be inhibited or even reversed through effective anti-fibrotic therapies (Kisseleva and Brenner, 2021; Pellicoro et al., 2014). However, no biological or chemical agents have been approved by the FDA for the treatment of liver fibrosis. Therefore, researching more effective novel therapies against liver fibrosis and focusing on early treatment are crucial steps in preventing and managing the severe complications associated with this condition.

Hepatic stellate cells (HSCs) are the primary fibrotic cell type in liver fibrosis, and the transformation of quiescent HSCs into myofibroblasts is central to the pathogenesis of liver fibrosis. Alpha-smooth muscle actin (α-SMA) is one of the key markers of HSCs activation (Kisseleva and Brenner, 2021). Continuous activation of HSCs is the driving force behind the progression of liver fibrosis, making the inhibition of HSC activation and proliferation a critical mechanism for reversing fibrosis. P2Y14, a purinergic G-protein coupled receptor, is expressed by a wide range of tissues and cell types, including immune cells, airway epithelium, brain, and the luminal and glandular epithelium of the endometrium (Liu et al., 2024; Kanamaru et al., 2024; Li et al., 2023; Karcz et al., 2021). It is involved in various cellular functions, such as immune regulation, vasoconstriction, and maintaining the stem cell compartment (Zhang et al., 2023). In a screening of damage-associated molecular pattern (DAMP) receptors, Mederacke’s team discovered that P2Y14 is highly enriched in HSCs, while its ligand, uridine 5′-diphosphate (UDP)-glucose, is abundant in hepatocytes and is released during different forms of cell death (Ray, 2022). Co-culturing HSCs with dying hepatocytes promoted HSC activation in a P2Y14-dependent manner. Both global and HSC-selective P2Y14 deficiency were found to attenuate liver fibrosis in multiple mouse models of liver injury (Mederacke et al., 2022). Thus, the P2Y14 receptor in HSCs is essential for the progression of liver fibrosis.

Qizhu-Ruogan-Granules (original name: Yiqi-Zhuyu-Jiedu-Granules; QZRG), a formulation from Hospital of the Chengdu University of TCM (Record No.: Sichuan Medicine Preparation Character Z20240014000), is composed of the classic recipe Gexia Zhuyu Decoction (Cao et al., 2022) combined with Xiaochaihu decoction (Wang et al., 2023), both of which have been verified by several experiments and have therapeutic effects on liver fibrosis and liver injury (Zeng et al., 2021; Hu et al., 2020; Zou et al., 2019; Deng et al., 2019; Chen et al., 2012), due to the unique humid and hot regional characteristics of the Sichuan Basin. It has been authorized by the national invention patent (patent number: ZL201610134757.9), which has been used as a fixed prescription in the clinical treatment of liver fibrosis for many years. Clinical studies have found that QZRG combined with antiviral drugs is effective in the treatment of patients with chronic hepatitis B liver fibrosis and cirrhosis with low-level viremia after long-term antiviral treatment (≥5 years), which the efficacy is better in patients with significant/progressive fibrosis than in patients with mild fibrosis, and no difference in efficacy due to different combination antiviral drugs (Dong et al., 2024). At the same time, previous animal experiments have shown that QZRG can effectively reduce the histological changes of liver fibrosis rats, reduce the serological indexes of liver fibrosis, inhibit the activation and proliferation of HSCs, and degrade excess extracellular matrix (ECM) (Hui et al., 2019a; Hui et al., 2019b; Yujing et al., 2024). In addition, in recent years, many advances have been made in the study of the mechanism of traditional Chinese medicine compounds in the treatment of liver fibrosis (Dong et al., 2023; Zhang and Schuppan, 2014; Liang et al., 2024; Fu et al., 2024; Xing et al., 2023; Ma et al., 2023; Gong et al., 2023; Dong et al., 2018). Therefore, it is of great significance to study the mechanism of QZRG on liver fibrosis disease and to further clarify the cell target of QZRG.

In this study, we investigated the role of P2Y14 in a mouse model of liver fibrosis induced by carbon tetrachloride (CCl_4_) and found that P2Y14 expression was significantly increased in liver fibrosis, showing the same trend as α-SMA. The expression of P2Y14 and α-SMA was decreased after the intervention of QZRG. Meanwhile, *in vitro* experiment, the expression of P2Y14 and α-SMA was decreased after the intervention of a P2Y14 inhibitor or QZRG. Although we found no evidence of the effect of QZRG on UDP-G, the expression of fibrotic protein in HSCs that inhibited while P2Y14 expression was significantly reduced. These results suggest that inhibiting the expression of P2Y14 on HSCs may be the target of QZRG to improve liver fibrosis.

## 2 Materials and methods

### 2.1 Reagents

#### 2.1.1 Preparation of experimental reagents

4-[4-(4-piperidinyl)phenyl]-7-[4-(trifluoromethyl)phenyl]2-naphthalenecarboxylic acid hydrochloride (PPTN; MedChemExpress, HY-110322), a selective antagonist for P2Y14, was prepared according to the manufacturer’s instruction. The carbon tetrachloride (CCl_4_, C805329) and olive oil (O815211) were purchased from Shanghai Macklin Biochemical Technology Co., LTD. (Shanghai, China). The primary antibodies of the experiment are P2Y14 Polyclonal Antibody (PA5-96964, Invitrogen, United States), Alpha smooth muscle actin Polyclonal antibody (14395-1-AP, Proteintech, Wuhan, China), Collagen Type I Polyclonal antibody (14695-1-AP, Proteintech, Wuhan, China), Collagen Type III (N-terminal) Polyclonal antibody (22734-1-AP, Proteintech, Wuhan, China), Caspase 3/p17/p19 Monoclonal antibody (66470-2-Ig, Proteintech, Wuhan, China), BAX Monoclonal antibody (60267-1-lg, Proteintech, Wuhan, China), Caspase 9/p35/p10 Monoclonal antibody (66169-1-lg, Proteintech, Wuhan, China), and Caspase 8/p43/p18 Monoclonal antibody (66093-1-lg, Proteintech, Wuhan, China). And internal reference antibodies are GAPDH Monoclonal antibody (60004-1-lg, Proteintech, Wuhan, China) and Beta Actin Monoclonal antibody (66009–1, Proteintech, Wuhan, China). Experimental operations were carried out according to the reagent instructions.

#### 2.1.2 Preparation of QZRG

Qizhu-Ruogan-Granules (QZRG) were obtained, authenticated, and made into granules by the Department of Pharmacy, Hospital of Chengdu University of Traditional Chinese Medicine (Chengdu, China). After measuring the weight of the whole decoction, add 8 times the amount of water, decocting and boiling for 3 times, each for 45 min. Combine the decoction and strain with 200 mesh gauze. The filtrate was condensed at 70°C to a thick paste with a relative density of 1.35 (60°C). Adjust the humidity of soft materials, and dry them into particles, packed in 15 g each. And the specific drug preparation process was shown in Supplementary Table S1. The original dosage of QZRG is shown in Table 1. And the plant names have been checked with Kew Medicinal Plant Names Services (MPNS) (Home page - Medicinal Plant Names Services ). The quality of the whole formula conforms to the standard of the Pharmacopoeia of the People’s Republic of China.

**TABLE 1:** The botanical drug(s) of QZRG.

Chinese name	Latin name	Origin	Weight	Batch number
Dangshen	*Codonopsis pilosula* (Franch.) Nannf.	Gansu	10 g	D2406006
Huangqi	*Astragalus mongholicus* Bunge	Gansu	15 g	D2407159
Ezhu	C*urcuma aromatica* Salisb.	Sichuan	10 g	2406071
Danggui	*Angelica sinensis* (Oliv.) Diels	Gansu	10 g	D2408155
Chuanxiong	*Conioselinum anthriscoides “Chuanxiong”*	Sichuan	5 g	2408210
Chishao	*Paeonia lactiflora* Pall.	Sichuan	10 g	2409045
Mudanpi	*Paeonia suffruticosa* Andr.	Anhui	10 g	2408137
Danshen	*Salvia miltiorrhiza* Bunge.	Sichuan	15 g	2407168
Chaihu	*Bupleurum chinensis* DC.	Sichuan	5 g	2407080
Huangqin	*Scutellaria baicalensis* Georgi	Shanxi	7.5 g	2408048
Zhike	*Citrus aurantium* L.	Sichuan	5 g	2409011
Gancao	*Glycyrrhiza uralensis* Fisch.	Neimengu	2.5 g	2407199

The particles were dissolved and diluted with 1:3 ratio of distilled water to produce 0.34 g/mL solution, centrifuged at 3000 RPM for 10 min, an ultrafiltration pump filtered the supernatant, the filtrate was collected and filtered by 0.22 um filter, and stored at −80°C for later use.

### 2.2 Design of animal experiment

Male wild-type C57BL/6J mice (16 ± 2 g) with 7 weeks old were purchased from SiPeiFu Biotechnology Co., LTD. (Beijing, China). For studies, mice were fed a normal diet and the experiment was started after 1 week of adaptive feeding. The Institutional Animal Ethics Committee of the Affiliated Hospital of Chengdu University of Traditional Chinese Medicine approved the study design with approval number 2024DL-001.

Mice were randomly divided into five groups: control (Veh + 0.9%NaCl), model (CCl_4_ + 0.9%NaCl), treatment of low (CCl_4_ + QZRG-low), treatment of middle (CCl_4_ + QZRG-middle), and treatment of high (CCl_4_ + QZRG-high). To induce fibrosis, male mice were intraperitoneally injected with olive oil or 10% CCl_4_ (dissolved in olive oil) at 5 μL/g of body weight, twice a week, from week 1 through week 15. The mice in the treatment groups were gavaged with QZRG (0.2 mL/10 g weight, once a day) for 8 weeks starting from the end of the seventh week. Adult daily dose of QZRG is 45 g/d The equivalent dose for C57BL/6J mice is about 9.1 times that of 70 kg adults, which means the daily dose for mice (1 kg) is about 5.85 g/d. The dose for QZRG-low is 2.925 g/(kg.d) of body weight, and 11.7 g/(kg.d) of body weight is just for QZRG-high. After 8 weeks of gavage, the mice were sacrificed, and blood and liver were collected for analysis. Mice were anesthetized by intraperitoneal injection of 0.3% pentobarbital sodium at 0.05 g/kg.

### 2.3 Cell culture

HSC-T6 cells (CL-0116), purchased from the Pricella Biotechnology Co., Ltd. (Wuhan, China), were cultured in DMEM supplemented with 1% penicillin/streptomycin and 10% FBS at 37°C in a 5% CO_2_ environment.

### 2.4 Solution preparation

#### 2.4.1 Determination of astragaloside content

For the astragaloside reference solution: The standards of astragaloside were accurately weighed, and methanol was added to dissolve them to prepare a solution with a solubility of 0.08168 mg/mL. The test solutions: Ground the QZRG finely, weighed 10 g precisely, added 140 mL of methanol, heated refluxes for 1 h, filtered, evaporated, dissolved the residue with 30 mL of water, extracted with n-Butanol saturated water, washed with the aqua ammonia and n-Butanol saturated water, evaporated. The residue was dissolved in 5 mL of methanol.

#### 2.4.2 Determination of seven other components including paeoniflorin

For the mixed reference solution: The reference substance 17.95 mg of calycosin-7-glucoside was accurately weighed and dissolved in methanol to 20 mL, as a reserve solution and stored in a refrigerator at 4°C–8°C. Precisely absorbed 1 mL of the reserve solution and diluted with methanol to 5 mL as the initial solution of the reference substance. The ammonium glycyrrhizinate reference substance 9.48 mg was accurately weighed, and the initial solution of the reference substance was prepared by the same method.

Next precisely weighed the reference substance salvianolic acid B 6.26 mg, baicalin 8.57 mg, neohesperidin 2.67 mg, naringin 3.11 mg, paeoniflorin 4.53 mg, then added 0.5 mL of calycosin-7-glucoside reference substance initial solution and 1 mL of ammonium glycyrrhizate reference substance initial solution, ultrasonic treatment (250 W, 40HZ) for 30 min, added methanol to 5 mL, as the No.6 reference substance mixed solution. Precisely absorbed 3 mL of the above No.6 reference mixed solution, diluted to 5 mL with methanol, as the No.5 reference mixed solution. The mixed solution of reference substances No.4, No.3, No.2, and No.1 was obtained in the same way.

The test solution: Ground the QZRG finely, accurately weighed 2 g of it, dissolved in methanol (concentration:80%) to 25 mL, treated with ultrasound (250 W/40 kHz) for 30 min, filtered, and obtained further filtrate. The reference substances are shown in Table 2.

**TABLE 2:** Reference substance.

Name	Batch number	Purity	Purchased from
Astragaloside reference substance	110781–201314	95.8%	The National Institutes for Food and Drug Control (Beijing, China)
Calycosin-7-Glucoside reference substance	111920–201314	98.3%	
Calycosin-7-Glucoside	111920–201606	97.6%	
Salvianolic acid B	111562–201716	94.1%	
Baicalin	110715–201720	93.5%	
Neohesperidin	11857–201703	99.2%	
Naringin	110722–201714	93.4%	
Ammonium glycyrrhizate	110731–201317	92.6%	
Paeoniflorin	110736–201438	96.4%	
Paeoniflorin	23180–57–6	98%	Chengdu Index Pure Biotechnology Co., LTD.

### 2.5 High performance liquid chromatography (HPLC) analysis for QZRG

#### 2.5.1 Content determination of astragaloside

HPLC with evaporative light scattering detector (HPLC-ELSD) determining astragaloside in QZRG, was established on an Agilent 1260 Infinity II LC System combined with an Agilent 1260 Infinity II Evaporative Light Scattering Detector (ELSD). A Phenomenex Luna C18 (2) column (4.6 × 250 mm, 5 μm, 100 A) was used to carry out separation alone with a mobile phase consisting of acetonitrile (A) and water (B). The gradient elution was as follows: 0–23 min, 33% (A); 24–28 min, 50% (A). The temperature of the column was 25°C, and the flow rate was 0.8 mL/min. The theoretical plate number should not be less than 4,000 according to the peak of astragaloside. Astragaloside reference solution (10 or 20 μL) and test solution (10 μL) were accurately absorbed and injected into liquid chromatograph for determination.

#### 2.5.2 Determination of seven other components including paeoniflorin

HPLC with diode array detector (HPLC-DAD) determining paeoniflorin, calycosin-7-glucoside, naringin, neohesperidin, baicalin, salvianolic acid B, and ammonium glycyrrhizate in QZRG, was established on an Agilent 1260 Infinity II LC System coupled to a diode array detector (DAD). A ZORBAX SB-C18 StableBond Analytical column (4.6 × 250 mm, 5 μm) was used to carry out the separation. The mobile phase consisted of a combination of A (acetonitrile) and B (0.2% phosphoric acid) with a linear gradient are shown in Table 3. The flow rate was 1.0 mL/min, the sample injection volume was 5 μL and the column temperature was 25°C. The diode array detector (DAD) was set at 237 nm for the real-time monitoring of the peak intensity.

**TABLE 3:** Gradient elution table.

Time/min	Mobile phase A/%	Mobile phase B/%
0	10	90
5	15	85
30	15	85
55	21	79
105	26	74
120	59	41

### 2.6 Histopathology examination

Mouse liver samples (the largest lobe of liver tissue) were collected and fixed at 4°C with 10% neutral formalin for 24 h. Fixed tissue specimens were embedded in paraffin and cut into 5 μm-thick tissue sections for histopathological analysis. Tissue sections were stained with Hematoxylin-Eosin (H&E) or Masson Trichrome according to standard instructions. The stained slides were examined using a light microscope by two experienced hepatologists blinded to the study protocol. The stage of liver fibrosis was assessed based on the following criteria: 0, normal, no fibrosis; 1, fibrosis present with collagen fibers extending from the portal triad/central vein to peripheral regions; 2, bridging fibrosis; 3, moderate fibrosis, portal-portal or portal-central linkage; and 4, definite cirrhosis (Desmet et al., 1994). The stage of liver fibrosis was also assessed based on the semiquantitative scoring system (SSS) criteria for liver fibrosis (Table 4) (Chevallier et al., 1994).

**TABLE 4:** Semiquantitative scoring system (SSS) criteria for liver fibrosis.

Score	Centrilobular vein (L)	Portal tract (P)	Number of septa (N)	Width of septa (W)
0	Normal or absence of vein (cirrhosis)	Normal	None	—
1	Moderately thickened	Enlarged without septa	≤6 septa/10 mm	Thin and/or incomplete
2	Markedly thickened	Enlarged with septa	>6 septa/10 mm	Thick and loose connective matrix
3	—	Cirrhosis	Nodular organization	Very thick and dense connective matrix
4	—	—	—	>2/3 biopsy area

Note: Score = L + P + 2 × (N × W), W score: if there is only one fine fiber interval, score 0.5, if the width of the fiber interval is between the two, take the average.

### 2.7 Biochemical testing

The serums were aspirated and stored at −80°C in the refrigerator. ALT, AST, and AKP were measured by the Japanese Olympus automatic biochemistry analyzer Au5400, and all data were finally exported.

### 2.8 Cell viability assay

Cells were seeded in 96-well plates (1.5 × 10^4^ cells/100 μL per well) with six duplications and then treated with QZRG for 48/72 h. Cell viability was measured using Enhanced Cell Counting Kit-8 (Beyotime, C0042, China) according to the manufacturer’s instructions. The absorbance was measured at 450 nm. Relative cell viability was calculated compared with control in each experiment.

### 2.9 HYP measurement by micromethod

The HYP levels of fresh liver samples of mice were quantified. Concentration was calculated by a standard curve using the HYP Content Assay Kit (Solarbio, BC0255, China) according to the manufacturer’s protocol.

### 2.10 UDP-G measurement by ELISA

The UDP-G levels of fresh liver samples of mice were quantified by using the Mouse UDP-G ELISA kit (CB11774-Mu, COIBO BIO, China) according to the manufacturer’s protocol.

### 2.11 Immunohistochemical staining

Immunohistochemical staining was performed according to the standard protocol. The primary antibody included Collagen Type I Polyclonal antibody (1:100), Collagen Type III (N-terminal) Polyclonal antibody (1:400), and Alpha smooth muscle actin Polyclonal antibody (1:800). The images obtained were observed under a microscope and the results were analyzed using Image-Pro Plus 6.0 software to export the data for data analysis.

### 2.12 Western blotting analysis

Western blot analysis was performed according to standard protocols. About 20 g of liver tissue was taken and fully decomposed by tissue lysis apparatus and liquid nitrogen. The protein lysates were separated by 10% sodium dodecyl sulphates polyacrylamide gel and then electrophoretically transferred (Bio-Rad) onto the PVDF membrane (Merck Millipore Ltd., Ireland). After blocking, membranes were incubated with the relevant primary antibodies P2Y14 Polyclonal Antibody (1:500), Alpha smooth muscle actin Polyclonal antibody (1:1,000), Collagen Type I Polyclonal antibody (1:1,000), Collagen Type III (N-terminal) Polyclonal antibody (1:1,000), Caspase 3/p17/p19 Monoclonal antibody (1:1000), BAX Monoclonal antibody (1:5,000), Caspase 9/p35/p10 Monoclonal antibody (1:500), and Caspase 8/p43/p18 Monoclonal antibody (1:2000) at 4°C overnight, and then were incubated with the secondary antibody goat anti-rabbit/mouse IgG antibody (1:5,000) at room temperature for 1 h, GAPDH Monoclonal antibody or Beta Actin Monoclonal antibody (1:5,000) for control.

### 2.13 RNA extraction and RT-qPCR assays

Total RNA was extracted from HSCs or liver tissue samples using MolPure^®^ Cell/Tissue Total RNA Kit (YEASEN, 119221ES50). For mRNA PCR, the total RNA was reverse transcribed to cDNA in a reaction volume of 20 μL using *Evo M-MLV* RT Mix Kit with gDNA Clean for qPCR Ver.2 (Accurate Biotechnology, AG11728). Quantitative real-time PCR reactions were carried out using SYBR^®^ Green Premix *Pro Taq* HS qPCR Kit (Rox Plus) (Accurate Biotechnology, AG11718) in a BIOER Gene-9660. The specific PCR primers are summarized in Table 5.

**TABLE 5:** PCR primers.

Gene	Forward (5′-3′)	Reverse (5′-3′)
GAPDH	AGG​TCG​GTG​TGA​ACG​GAT​TTG	TGT​AGA​CCA​TGT​AGT​TGA​GGT​CA
P2y14	AGC​AGA​TCA​TTC​CCG​TGT​TGT	AGC​CAC​CAC​TAT​GTT​CTT​GAG​G
α-SMA	GTC​CCA​GAC​ATC​AGG​GAG​TAA	TCG​GAT​ACT​TCA​GCG​TCA​GGA
COL1	GCT​CCT​CTT​AGG​GGC​CAC​T	CCA​CGT​CTC​ACC​ATT​GGG​G
COL3	CTG​TAA​CAT​GGA​AAC​TGG​GGA​AA	CCA​TAG​CTG​AAC​TGA​AAA​CCA​CC

### 2.14 Statistics

The number of independent experiments and the statistical analysis for each figure are indicated in the legends. All statistical analyses were performed using GraphPad Prism version 9 for Windows (GraphPad Software, La Jolla California United States, www.graphpad.com) and are presented as mean ± SEM. Comparisons among groups were done using one-way ANOVA with Tukey post-test. *P* < 0.05 was considered significant.

## 3 Results

### 3.1 Identification of the chemical metabolite(s) of QZRG granules by HPLC-ELSD and HPLC-DAD

**FIGURE 1:**
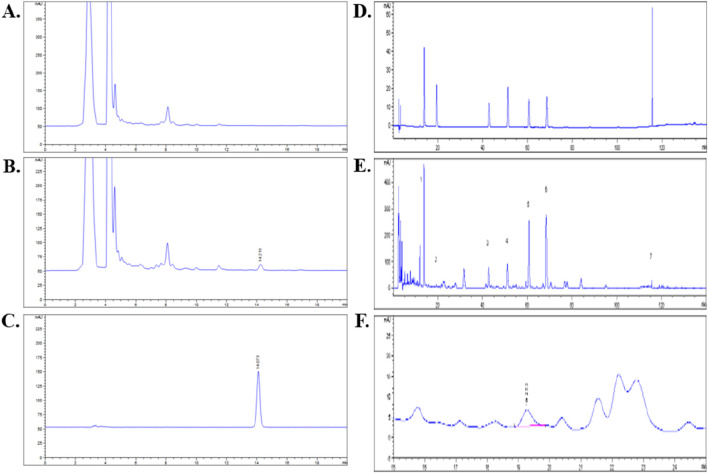
HPLC analysis for QZRG: **(A)** HPLC chromatogram of QZRG except for Huangqi. **(B)** HPLC chromatogram of the QZRG. **(C)** HPLC chromatogram of astragaloside reference substance. **(D)** HPLC chromatograms of seven mixed reference substances. **(E)** HPLC chromatogram of the QZRG. **(F)** The enlarged chromatogram of calycosin-7-glucoside in the chromatogram of QZRG. The values marked in **(E)** are represented as follows:1: Paeoniflorin, 2: Calycosin-7-Glucoside, 3: Naringin, 4: Neohesperidin, 5: Baicalin, 6: Salvianolic acid B, 7: Ammonium glycyrrhizate.

Eight metabolite(s) were identified in QZRG granules. Astragaloside in QZRG was determined by HPLC-ELSD. The granules contained 0.0413 mg astragaloside per gram, the HPLC chromatograms shown in Figures 1A–C. Paeoniflorin, Calycosin-7-Glucoside, naringin, neohesperidin, baicalin, salvianolic acid B, and ammonium glycyrrhizate in QZRG were determined by HPLC-DAD, the representative metabolite(s) shown in Figures 1D–F and Table 6.

**TABLE 6:** Contents of eight metabolite(s) in QZRG (mg/g).

Metabolite(s)	Contents
Astragaloside	0.0413 mg
Paeoniflorin	3.5613 mg
Calycosin-7-Glucoside	0.0748 mg
Naringin	2.8719 mg
Neohesperidin	3.1133 mg
Baicalin	8.217 mg
Salvianolic acid B	5.2097 mg
Ammonium glycyrrhizate	0.1737 mg

### 3.2 QZRG granules alleviate CCl_4_-induced liver fibrosis in mice

**FIGURE 2:**
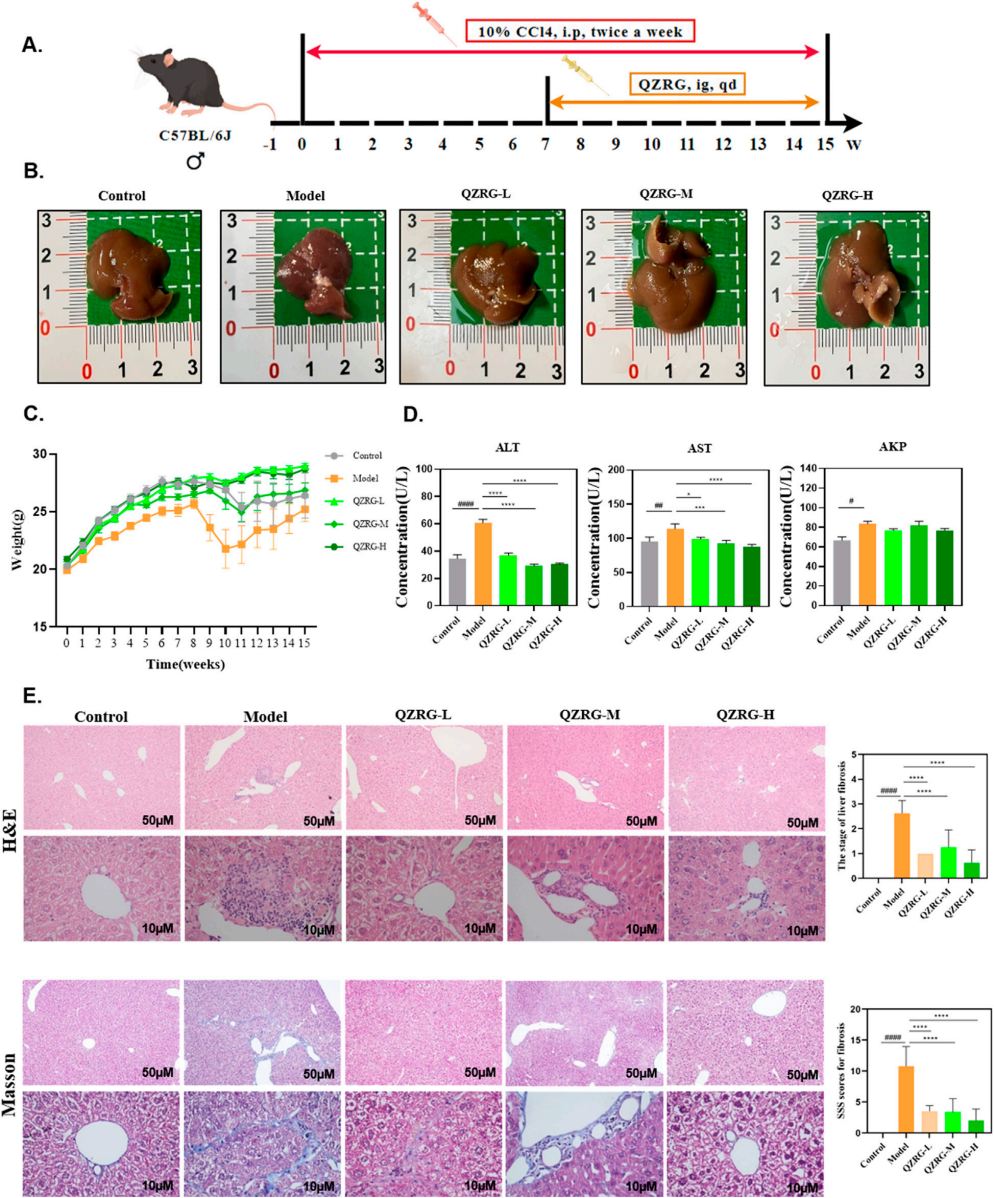
QZRG granules alleviate CCl_4_-induced liver fibrosis in mice. **(A)** Liver fibrosis modeling: Experimental modeling process and intervention. After 7 weeks of intraperitoneal injection, the model was successfully established, then the drug gavage treatment lasted for 8 weeks. **(B)** Macroscopic liver phenotype (n = 8). **(C)** Trend of body weight change in mice (n = 8 mice per group). **(D)** Liver laboratory values on ALT, AST, and AKP (n = 8 mice per group). **(E)** H&E and Masson staining in the liver tissues of mice from different groups (n = 8 mice per group, ×100: scale bar = 50 μm, ×400: scale bar = 10 μm). Data are presented as mean ± SEM. #P < 0.05, ##P < 0.01, ###P < 0.001, ####P < 0.0001 (vs. Control group); *P < 0.05, **P < 0.01, ***P < 0.001, ****P < 0.0001 (vs. Model group); One-way analysis of variance (One-way ANOVA), the growth differences between control and model, and model with QZRG-L, QZRG-M, or QZRG-H were compared. AST: aspartate aminotransferase, ALT: alanine aminotransferase, AKP: serum alkaline phosphatase.

Liver toxicity induced by CCl_4_ results in severe liver cell damage, eventually progressing to liver fibrosis. To assess whether QZRG granules could alleviate liver damage, mice were intraperitoneally injected with 10% CCl_4_ for 8 weeks, and from week 7 onwards, they were administered daily doses of QZRG granules by gavage (Figure 2A). At the end of the experiment, overall liver condition and mouse body weight were recorded, and serum levels of aspartate aminotransferase (AST), alanine aminotransferase (ALT), and serum alkaline phosphatase (AKP) were measured. Following data recording and analysis, it was observed that the QZRG granule treatment group, as shown in Figures 2B, C, effectively improved the liver tissue condition and mitigated weight loss in the mice. In parallel, Figure 2D shows that ALT, ALP, and AKP levels were significantly elevated in the CCl_4_-treated group compared to the control group, highlighting that these markers, critical for liver function, indicated impaired liver function due to carbon tetrachloride exposure. However, treatment with QZRG granules significantly reduced ALT, ALP, and AKP levels across all treatment groups. Histological analysis of liver sections using H&E and Masson staining revealed that with increasing QZRG granule doses, there was a significant reduction in inflammatory cell infiltration and a marked decrease in liver fibrosis SSS scores (Figure 2E). Collectively, these data demonstrate that QZRG granules effectively alleviate CCl_4_-induced liver fibrosis and enhance liver function.

### 3.3 QZRG granules reduce collagen accumulation and inhibit hepatic stellate cell activation in CCl_4_-induced liver fibrosis

**FIGURE 3:**
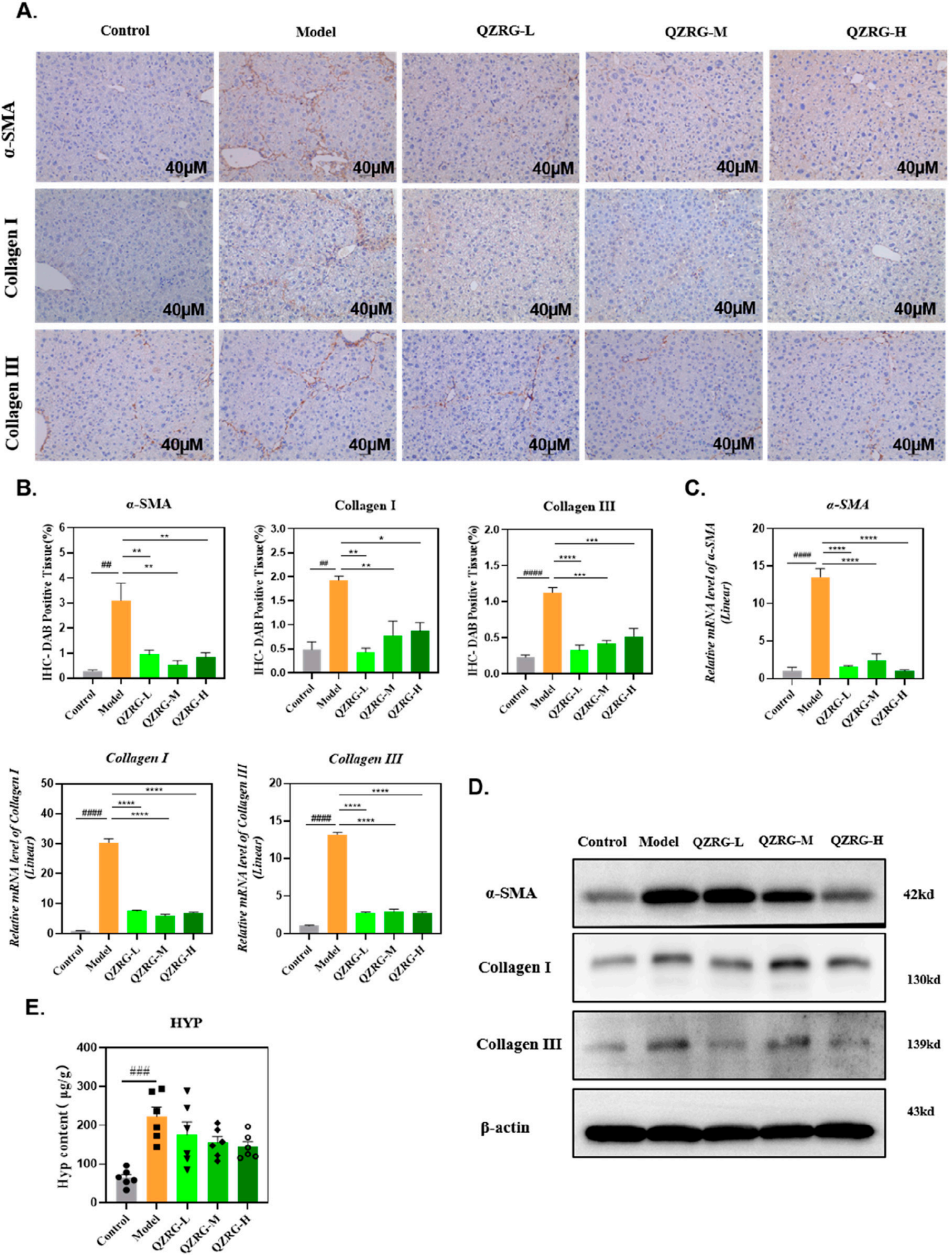
QZRG granules reduce collagen accumulation and inhibit hepatic stellate cell activation in CCl_4_-induced liver fibrosis. **(A)** The immunohistochemistry images of liver tissues stained with α-SMA, COL1, and COL3 in different groups (n = 3 mice per group, scale bar = 40 μm, 20×). **(B)** The DAB-positive tissue (%) in immunohistochemistry images of liver tissues stained with α-SMA, COL1, and COL3 in different groups (n = 3 mice per group). **(C)** The effect of QZRG on the mRNA expression level of α-SMA, COL1, and COL3 in liver tissues of mice analyzed by Real-time PCR. **(D)** The effect of increasing concentration of QZRG on the protein levels of α-SMA, COL1, and COL3 in liver tissues of mice by Western blot. **(E)** Hydroxyproline (Hyp) content in liver tissues was measured (n = 6 mice per group). Data are presented as mean ± SEM. #P < 0.05, ##P < 0.01, ###P < 0.001, ####P < 0.0001 (vs. Control group); *P < 0.05, **P < 0.01, ***P < 0.001,****P < 0.0001 (vs. Model group); one-way ANOVA with Tukey’s *post hoc* test, the growth differences between control and model, and model with QZRG-L, QZRG-M, or QZRG-H were compared.

We next investigated the ability of QZRG granules to downregulate collagen expression in the liver. Immunohistochemistry was employed to assess collagen deposition and α-SMA expression in liver sections, and the results were analyzed. As shown in Figures 3A, B, the CCl_4_-treated group demonstrated significant Collagen I (COL1) and Collagen III (COL3) deposition along with elevated α-SMA levels, compared to the control group. In contrast, treatment with QZRG granules significantly reduced collagen fiber accumulation and α-SMA expression, suggesting that QZRG granules may mitigate fibrosis by reducing collagen deposition and inhibiting hepatic stellate cell activation.

In addition, we evaluated the protein and mRNA expression levels of α-SMA, COL1, and COL3 in the liver tissues of each group through Western blotting and quantitative reverse transcription PCR (RT-PCR). As illustrated in Figures 3C, D, QZRG granules markedly reduced the CCl_4_-induced elevation of α-SMA, COL1, and COL3 at both the mRNA and protein levels. Furthermore, we measured hydroxyproline (HYP), a key component of collagen, and as shown in Figure 3E, the HYP results were consistent with the collagen mRNA and protein levels. Collectively, these findings suggest that QZRG granules significantly inhibit hepatic stellate cell activation and reduce the elevated collagen levels in the liver tissues of CCl_4_-treated mice.

### 3.4 QZRG granules decreased the expression level of P2Y14 in CCl_4_-induced liver fibrosis

**FIGURE 4:**
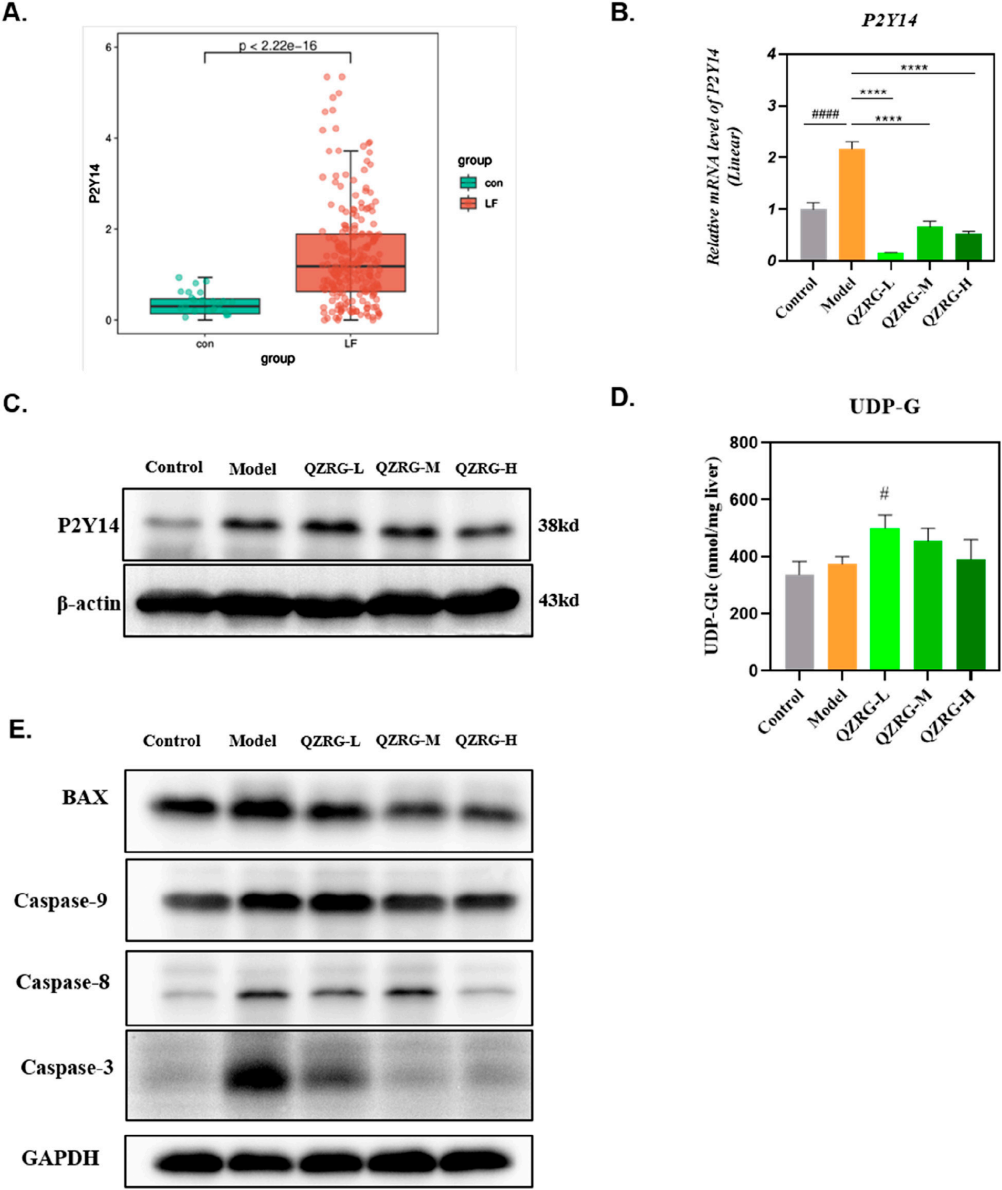
QZRG granules decreased the expression level of P2Y14 in CCl_4_-induced liver fibrosis. **(A)** P2Y14 expression was different in 27 normal liver tissue samples and 216 fibrosis tissue samples. **(B)**The effect of QZRG on the mRNA expression level of α-SMA in liver tissues of mice analyzed by Real-time PCR. The experiment was performed in triplicates and data are presented as mean ± SEM. #P < 0.05, ##P < 0.01, ###P < 0.001, ####P < 0.0001 (vs. Control group); *P < 0.05, **P < 0.01, ***P < 0.001, ****P < 0.0001 (vs. Model group). **(C)** The effect of increasing concentration of QZRG on the protein levels of P2Y14 in liver tissues of mice by Western blot. **(D)** The content of UDP-G in liver tissues of mice in different groups (n = 6 mice per group). **(E)** The expression levels of apoptosis-related proteins BAX, Caspase9, Caspase8, and Caspase3 in liver tissues of mice by Western blot. Data are presented as mean ± SEM. #P < 0.05, ##P < 0.01, ###P < 0.001, ####P < 0.0001 (vs. Control group); *P < 0.05, **P < 0.01, ***P < 0.001, ****P < 0.0001 (vs. Model group); One-way analysis of variance (One-way ANOVA), the growth differences between control and model, and model with QZRG-L, QZRG-M, or QZRG-H were compared.

To determine the expression level of P2Y14 in liver fibrosis, we obtained the GSE263786 dataset from the GEO database (https://www.ncbi.nlm.nih.gov/geo/), which includes 27 normal liver tissue samples and 216 liver fibrosis tissue samples. The data was cleaned using R software, and the count expression matrix was converted into a TPM expression matrix. Subsequently, the expression levels of P2Y14 were extracted. The R packages “ggplot2” and “ggsignif” were used to analyze the differences in P2Y14 expression and generate a box plot. As shown in Figure 4A, P2Y14 expression was significantly upregulated in liver fibrosis tissue samples. Moreover, we found that both the mRNA and protein expression levels of P2Y14 were markedly increased in the liver tissues of mice with CCl_4_-induced liver fibrosis, while these levels were significantly reduced following QZRG granule treatment (Figures 4B, C). Based on these results, we hypothesized that QZRG granules might treat liver fibrosis by modulating P2Y14 expression. To further explore this, we measured the content of UDP-G in the samples. However, as shown in Figure 4D, the expression level of UDP-G did not change in the CCl_4_-induced fibrosis model, and QZRG granules had no effect on it. This suggests that QZRG granules may exert their therapeutic effect on liver fibrosis through other mechanisms involving P2Y14.

It has been previously reported that P2Y14 ligands or signals from dead liver cells can activate HSCs by stimulating the P2Y14 receptor (Mederacke et al., 2022). Therefore, we speculated that QZRG granules might regulate P2Y14 expression by modulating the overall apoptosis level in liver tissue, thereby contributing to the treatment of liver fibrosis. To test this, we assessed the overall apoptosis levels in liver tissues from the experimental mice. As shown in Figure 4E, the expression levels of apoptosis-related proteins BAX, Caspase9, Caspase8, and Caspase3 were all reduced in the liver tissues of mice with CCl_4_-induced liver fibrosis.

### 3.5 QZRG granules inhibit the expression of P2Y14 in activated hepatic stellate cells and promote the apoptosis of these activated cells

**FIGURE 5:**
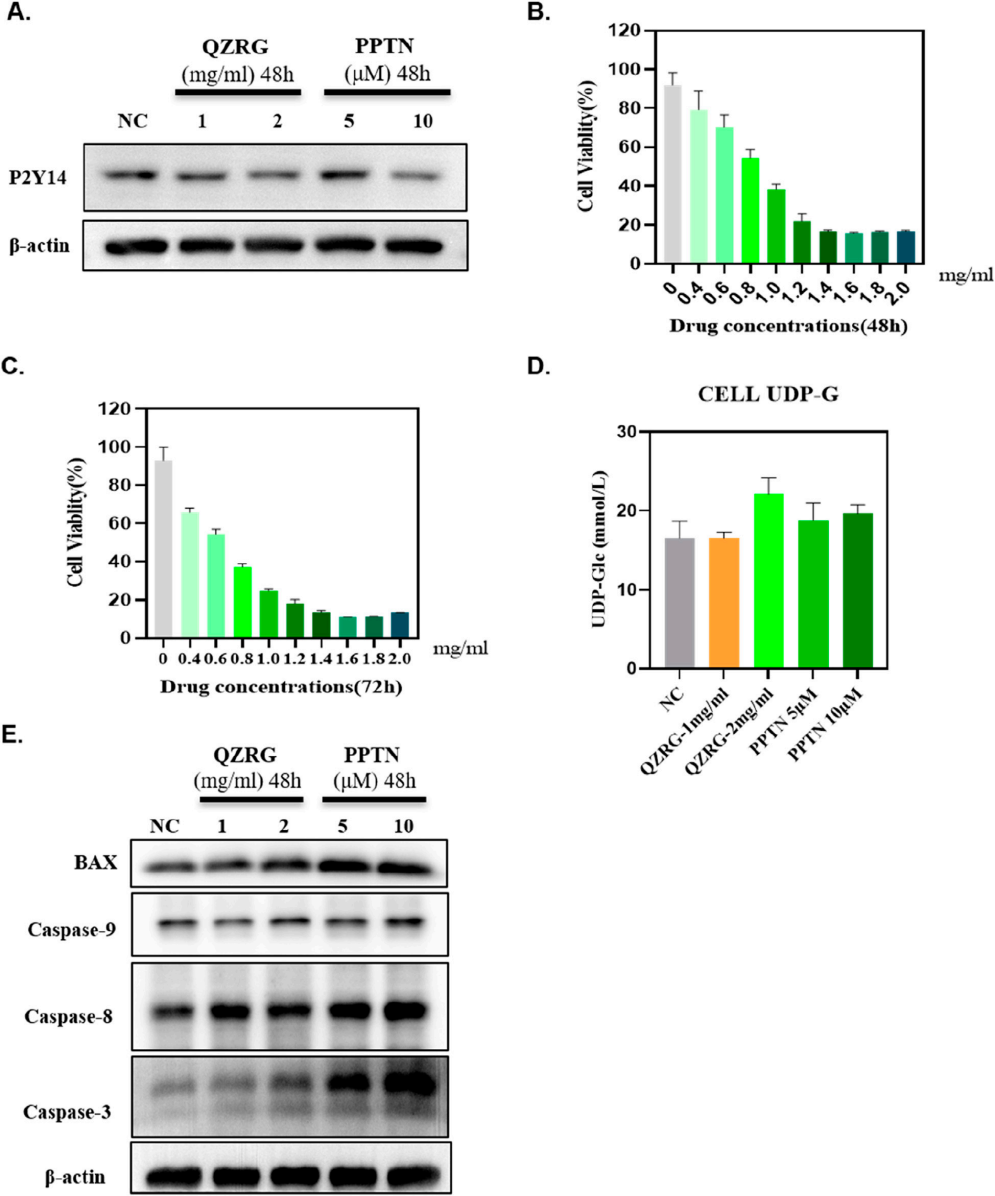
QZRG granules inhibit the expression of P2Y14 in activated hepatic stellate cells and promote the apoptosis of these activated cells. **(A)** The expression of P2Y14 in HSC-T6 cells after 48 h of treatment with water extracts of QZRG granules or PPTN by Western blot. **(B, C)** The cell viability of HSC-T6 cells after 48/72 h of treatment with water extracts of QZRG granules was measured using Enhanced Cell Counting Kit-8. **(D)** The content of UDP-G in HSC-T6 cells with water extracts of QZRG granules or PPTN. Data are presented as mean ± SEM. **(E)** The expression levels of apoptosis-related proteins BAX, Caspase9, Caspase8, and Caspase3 in HSC-T6 cells after 48 h of treatment with water extracts of QZRG granules or PPTN by Western blot.

The *in vivo* experiment results demonstrated that QZRG granules significantly reduced overall apoptosis levels in the liver tissues of mice with liver fibrosis and markedly decreased the expression of P2Y14. Based on this, we investigated whether P2Y14 expression in activated hepatic stellate cells (HSCs) was consistent with that in liver tissues and whether it affected apoptosis levels in HSCs. Therefore, we stimulated activated HSC-T6 cells with water extracts of QZRG granules, using a P2Y14 inhibitor as a control. As shown in Figure 5A, after 48 h of treatment, QZRG granules significantly inhibited P2Y14 expression in HSC-T6 cells. Additionally, as demonstrated in Figures 5B, C, we observed that QZRG granules also suppressed HSC-T6 cell proliferation. However, as illustrated in Figure 5D, QZRG granules did not alter UDP-G levels in HSC-T6 cells, a result consistent with observations in liver tissues. Therefore, we hypothesize that QZRG granules do not affect P2Y14 expression by modulating UDP-G levels. Based on these results, we hypothesized that QZRG granules inhibit HSC-T6 cell proliferation by promoting apoptosis. To verify this, we measured apoptosis-related protein expression levels after QZRG granule treatment. As shown in Figure 5E, QZRG granules increased apoptosis levels in HSC-T6 cells, similar to the effect of the P2Y14 inhibitor. Consequently, we suggest that QZRG granules promote apoptosis in HSC-T6 cells by downregulating P2Y14 expression, thereby inhibiting cell proliferation.

## 4 Discussion

The CCl_4_-induced animal liver fibrosis model is a well-established and reliable tool for studying human liver fibrosis. Using this model, we demonstrated that QZRG granules effectively alleviate CCl_4_-induced liver fibrosis. Specifically, QZRG granules reduce collagen accumulation and inhibit the mRNA and protein levels of α-SMA, a marker of hepatic stellate cell activation. Furthermore, QZRG granules suppressed the mRNA and protein levels of collagen types I and III (COL1, COL3). Continuous hepatocyte injury and repair contribute to increased inflammatory cell infiltration and the release of pro-inflammatory cytokines, key factors driving liver fibrosis. By assessing liver injury markers—alanine aminotransferase (ALT) and aspartate aminotransferase (AST)—we found that QZRG granules mitigated CCl_4_-induced liver injury.

Fibrosis is a major determinant of outcomes in chronic liver disease, yet effective anti-fibrotic therapies remain scarce. Although platelet-derived growth factor and transforming growth factor-β are recognized as key mediators of fibrosis, they do not fully elucidate the relationship between hepatocyte death and fibrosis. Previous studies have highlighted the role of apoptotic cell engulfment in hepatic stellate cell (HSC) activation. Additionally, research has shown that hepatocyte apoptosis can activate P2Y14, thereby promoting HSC activation (Canbay et al., 2003; Zhan et al., 2006). Our findings demonstrate that QZRG granules exert a therapeutic effect on liver fibrosis by inhibiting overall apoptosis levels in liver tissues of fibrotic mice, which in turn leads to decreased P2Y14 expression. Based on these results, we further explored whether QZRG granules also influence the state of activated HSCs. To this end, we treated activated HSC-T6 cells with water extracts of QZRG granules, using a P2Y14 inhibitor as a control. Our results revealed that QZRG granules inhibited P2Y14 expression in activated HSCs and suppressed their proliferation. In liver tissues, QZRG granules reduced overall apoptosis levels in CCl_4_ model mice. However, our findings showed the opposite effect in activated HSCs, where QZRG granules promoted apoptosis. Since liver tissue comprises multiple cell types, and HSCs are only one component, we speculate that QZRG granules may have different effects on activated HSCs and other liver cells. QZRG granules not only inhibit normal hepatocyte apoptosis but also promote the apoptosis of activated stellate cells.

## 5 Conclusion

Our results indicate that QZRG granules can reduce liver damage and inflammation in CCl_4_-induced mouse liver models, while also inhibiting collagen accumulation. Additionally, QZRG granules downregulated P2Y14 expression by reducing overall apoptosis levels in liver tissues of CCl_4_ model mice. Furthermore, they promoted apoptosis in activated hepatic stellate cells by inhibiting P2Y14 expression within these cells. In conclusion, as a drug that has been used clinically for many years, the therapeutic efficacy of QZRG granules against liver fibrosis has been well established in clinical treatments. Our data suggest that QZRG granules may exert antifibrotic effects by inhibiting P2Y14 expression, thus shedding light on the underlying mechanism of QZRG granules.

## Data Availability

The original contributions presented in the study are included in the article/Supplementary Material, further inquiries can be directed to the corresponding authors.
